# Cutaneous malignancies mimicking scars: A case series highlighting pearls and pitfalls

**DOI:** 10.1016/j.jdcr.2025.11.038

**Published:** 2025-12-05

**Authors:** Jordan Hill, Brooke A. Burgess, Chad M. Hivnor, Joshua L. Owen

**Affiliations:** a Long School of Medicine, University of Texas Health Science Center San Antonio, San Antonio, Texas; b Division of Dermatology, University of Texas Health Science Center San Antonio, San Antonio, Texas; c Dermatology Service, South Texas Veterans Health Care System, San Antonio, Texas

**Keywords:** cutaneous leiomyosarcoma, dermatofibrosarcoma protuberans, desmoplastic melanoma, mimics, sarcomatoid squamous cell carcinoma, scars

## Introduction

Scars and cutaneous lesions that resemble scars are frequently encountered in dermatologic practice and are often presumed to be benign. However, a minority of these lesions either represent or mask malignant neoplasms. The clinical similarity between scars and scar-like malignancies can lead to a delay in accurate diagnosis, definitive treatment, and potentially increased patient morbidity.

In this case series, we describe 4 cases in which a lesion clinically mimicked a scar, but given certain clinical features and a high index of suspicion, a biopsy was performed, leading to a malignant diagnosis, including desmoplastic melanoma, dermatofibrosarcoma protuberans (DFSP), leiomyosarcoma, and sarcomatoid squamous cell carcinoma (SCC).

## Cases

### Case 1

**Fig 1:**
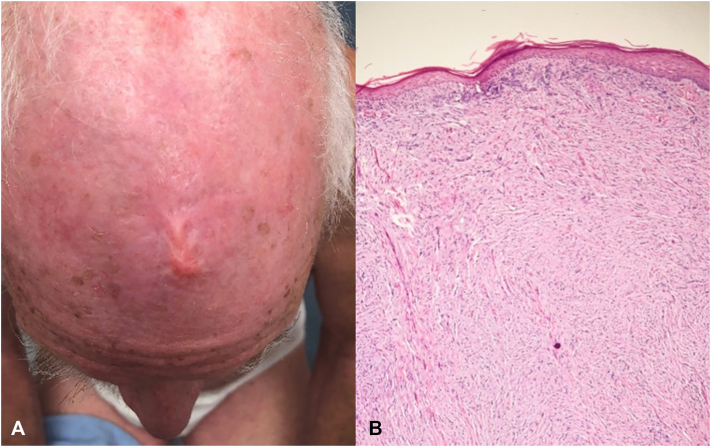
Desmoplastic melanoma. **A,** Clinical photograph of a 1.2 × 1.2 cm erythematous, indurated, smooth plaque of the frontal scalp on initial presentation. **B,** Excisional biopsy histopathology depicting haphazard atypical spindle cells with elongated nuclei in a dense, fibrous stroma (hematoxylin-eosin stain; original magnification: 10×).

A 78-year-old male patient with a history of previous keratinocyte carcinomas, including those of the scalp, presented with concern for a “scar” on his frontal scalp. Physical examination revealed a 1.2 × 1.2 cm skin-colored, smooth, indurated plaque (Fig 1, *A*). A shave biopsy was performed; pathology revealed dermal fibrosis consistent with scar. Per the patient’s preference, the lesion was treated with intralesional triamcinolone (ILTAC). Eight months later, the lesion had grown significantly to a 2.5 × 2.5 cm eroded, exophytic plaque. A second shave biopsy (wider and deeper than previously) was performed; pathology revealed atypical spindle cell neoplasm in the dermis. Immunohistochemical stains revealed SOX-10 and S-100 positivity, with negative staining of MART-1, p63, and desmin, consistent with desmoplastic melanoma. Excisional biopsy was then performed, revealing Breslow depth of 1.1 cm and foci suspicious for perineural and lymphovascular invasion (Fig 1, *B*). The patient underwent a sentinel lymph node biopsy and wide local excision (WLE) by otolaryngology with no lymph node involvement and negative surgical margins.

### Case 2

**Fig 2:**
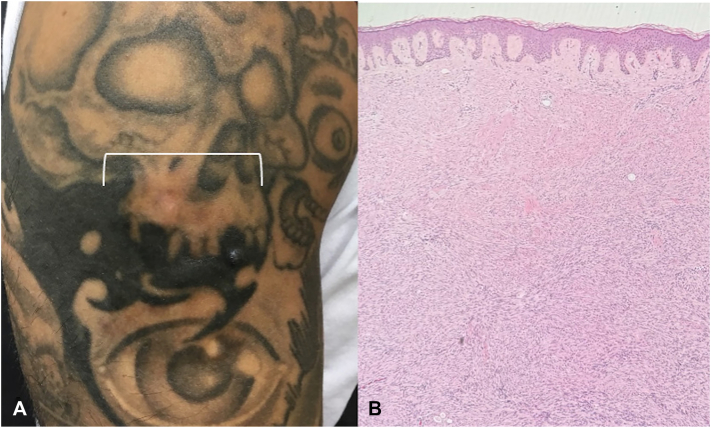
Dermatofibrosarcoma protuberans. **A,** Clinical photograph of 4.0 × 4.0 cm subcutaneous, indurated plaque of the lateral aspect of the upper portion of the right arm within a tattoo (white bracket). **B,** Shave biopsy histopathology with dermal proliferation of wavy, spindle-shaped cells extending throughout dermis (hematoxylin-eosin stain; original magnification: 4×).

A 39-year-old male patient with a history of ear keloids 5 years prior and no past medical history of cutaneous malignancy presented to the clinic out of concern for a hard nodule of the right arm within a tattoo (he denied other trauma to the area). Physical examination revealed a 4.0 × 4.0 cm smooth, indurated, slightly erythematous plaque of the lateral aspect of the upper portion of the right arm (Fig 2, *A*). Differential diagnosis included scar, cutaneous sarcoidosis, granulomatous tattoo reaction, or morphea. Given the possibility of a deeper pathologic component, a deep shave biopsy was performed; pathology revealed densely hypercellular wavy and spindle-shaped CD34^+^, factor XIIIa cells in a storiform pattern infiltrating underlying fat with no evidence of fibrosarcomatous transformation (Fig 2, *B*). Diagnosis of DFSP was made. Magnetic resonance imaging of the right upper extremity revealed a 6.5 × 4.4 cm soft tissue mass, measured 8.1 mm in depth, with extension to the superficial fascial plane of the deltoid without definitive invasion into musculature. The patient underwent WLE with clear surgical margins by surgical oncology and subsequent placement of split-thickness skin graft by plastic surgery.

### Case 3

**Fig 3:**
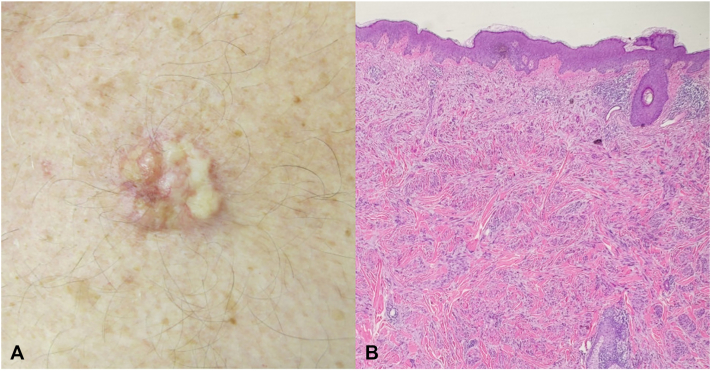
Cutaneous leiomyosarcoma. **A,** Clinical photograph of a 1.5 × 1.5 cm erythematous multinodular plaque on the left side of the chest. **B,** Excisional biopsy histopathology depicting poorly circumscribed dermal proliferation of eosinophilic spindle cells with blunt-ended nuclei (hematoxylin-eosin stain; original magnification: 4×).

A 59-year-old male with a past medical history of multiple keratinocyte carcinomas of the head and neck presented with a lesion on his chest. He reported that the lesion was biopsied 5 years ago by another clinician, who histologically diagnosed it as a keloidal scar. Physical examination revealed a 1.5 × 1.5 cm erythematous, indurated, mamillated plaque on the left side of the chest (Fig 3, *A*). The lesion appeared clinically consistent with a keloidal scar and was treated with ILTAC and intralesional 5-fluorouracil. After 4 treatments of increasing concentrations of ILTAC in a 1:1 ratio with 5-fluorouracil, the lesion had increased in size and remained tender. An excisional biopsy was performed; pathology revealed a highly cellular proliferation of fusiform cells with eosinophilic cytoplasm that stained positively with smooth muscle actin and desmin, consistent with leiomyosarcoma (Fig 3, *B*). Magnetic resonance imaging of the chest with and without contrast revealed a plate-like thickening of the dermis measuring 7.0 cm in width and 0.45 cm thick with underlying subcutaneous fat stranding and no evidence of gross underlying muscular or perineural invasion. Mohs micrographic surgery was performed, and complete histologic clearance was achieved after 1 stage.

### Case 4

**Fig 4:**
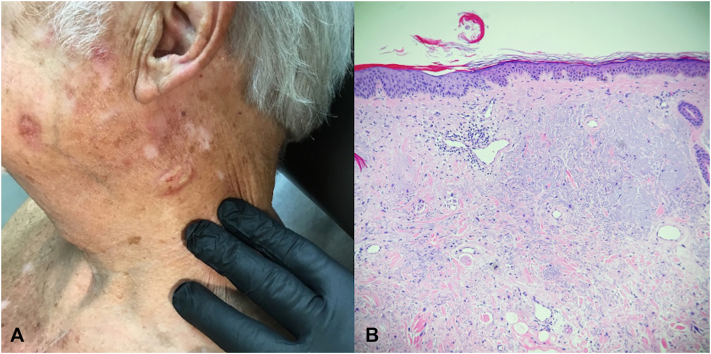
Sarcomatoid squamous cell carcinoma. **A,** Clinical photograph of a 1.5 × 0.8 cm skin-colored, smooth plaque of the left lateral aspect of the neck. **B,** Shave biopsy histopathology depicting poorly differentiated, spindle cell proliferation in the dermis with pronounced solar elastosis and thickened collagen bundles (hematoxylin-eosin stain; original magnification: 4×).

A 75-year-old male patient with a history of multiple keratinocyte carcinomas presented with a progressively growing 1.5 × 0.8 cm skin-colored, smooth plaque of the left lateral aspect of the neck (Fig 4, *A*). A partial shave biopsy was performed, and the pathology was significant for solar elastosis with a superficial dermal scar. Three months later, the patient returned to the clinic stating the residual lesion was continuing to grow. A second shave biopsy (larger and deeper) was performed and initially diagnosed as a scar again, but upon second opinion, revealed a pleomorphic spindle cell proliferation between collagen bundles (Fig 4, *B*) that stained positive with cytokeratins AE1/3, consistent with sarcomatoid SCC. WLE was performed by surgical oncology with negative margins.

## Discussion

Skin and soft tissue malignancies can vary widely in clinical presentation and have the potential to mimic benign lesions such as scars. This case series highlights 4 cases in which a lesion clinically presented as a scar (2 of which were initially diagnosed as scar histopathologically) but was subsequently found to be a malignant tumor, including desmoplastic melanoma, DFSP, leiomyosarcoma, and sarcomatoid SCC. Although this list of scar-mimicking lesions is not exhaustive, it illustrates the variety of skin malignancies that can be encountered in scar-like lesions.

Cutaneous malignancies may be difficult to distinguish from scars by physical examination alone because both entities can appear skin-colored, smooth, and indurated, with slow growth and tenderness. This is particularly true when the malignancy is primarily in the dermis or deeper, as seen in the 4 cases presented. A subtle presentation and lack of rapid growth or ulceration can lead to the selection of ineffective treatment modalities, resulting in delayed diagnosis/definitive treatment. When more superficial skin structures are involved (epidermis, ulceration), lesions tend to raise clinical concerns sooner.

Therefore, a low threshold for biopsy during evaluation of scar-like lesions is imperative, and biopsy technique is an important consideration. Many spindle cell tumors have bland histology, are deeply infiltrative, and can occur concomitantly with a true scar. Thus, a superficial biopsy might result in a sampling error and reveal only an overlying scar. Given potential diagnostic difficulty, we recommend using a technique that will allow for histopathologic assessment to the level of the adipose (eg, an excisional biopsy or a standard or telescoping punch biopsy) as performed in cases 2 and 4. Many scar–mimicking skin malignancies exhibit key diagnostic features in the deep dermal and superficial adipose layers, necessitating inclusion of this tissue to arrive at a correct diagnosis.

Immunohistochemistry is an invaluable tool that can aid in distinguishing scars from scar-like malignancies on histopathology, particularly those composed of spindle cells within a fibrotic stroma. As many of these neoplasms can exhibit bland cytology, minimal pleomorphism, and overlapping characteristics with benign fibrous processes, immunohistochemistry serves to distinguish between these entities. For example, desmoplastic melanoma typically lacks expression of melanocytic markers such as MART-1 and HMB-45 but stains positively for S-100 and SOX-10.^1^^,^^2^ Sarcomatoid SCC typically lacks classic squamous differentiation but can be identified as epithelial in origin by cytokeratins AE1/3.^3^ While often less histopathologically subtle, DFSP can be identified by CD34 positivity and factor XIIIa negativity, distinguishing it from dermatofibromas and other spindle cell tumors.^4^ Finally, leiomyosarcoma stains positively for both smooth muscle actin and desmin, confirming muscular origin.^5^

These cases highlight 4 key principles. First, a broad differential diagnosis should be retained for any lesion clinically resembling a scar, especially in the absence of a clear history of trauma. Second, lesions that fail to respond to standard treatment, such as intralesional steroids or 5-fluorouracil, should undergo prompt histopathologic evaluation or reevaluation. Additionally, the biopsy technique is critical; partial or superficial sampling may fail to adequately assess deep, paucicellular tumors. Therefore, deeper biopsy techniques that include the dermal-adipose junction offer a higher diagnostic yield. Early and accurate diagnosis of cutaneous malignancy is essential to avoid delayed treatment and to reduce patient morbidity or mortality. Finally, given the complexity and aggressive/invasive behavior of these malignancies, multidisciplinary care is often necessary to provide the most appropriate and effective treatment.

In conclusion, this case series highlights the importance of remaining vigilant when evaluating and treating scar–like cutaneous lesions, particularly those that appear atypical or lack response to standard treatment modalities. Clinical-pathologic correlation through use of appropriate biopsy technique and histopathologic evaluation with immunohistochemical markers when indicated are vital to avoid misdiagnosis and ensure optimal patient outcomes.

## Conflicts of interest

None disclosed.
